# P-842. Implementing a Tailored Prescription Strategy for Outpatient Sinusitis Management

**DOI:** 10.1093/ofid/ofaf695.1050

**Published:** 2026-01-11

**Authors:** Christen J Arena, Rachel M Kenney, Susan L Davis, Anita Shallal, John R Craig, Michael P Veve

**Affiliations:** University of Cincinnati, Cincinnati, OH; Henry Ford Hospital, Detroit, Michigan; Wayne State University, Detroit, Michigan; Henry Ford Hospital, Detroit, Michigan; Henry Ford Hospital, Detroit, Michigan; Eugene Applebaum College of Pharmacy and Health Sciences, Detroit, MI

## Abstract

**Background:**

Sinusitis is a leading cause for outpatient antibiotic use. Internal data suggests that when antibiotics are prescribed for sinusitis, the preferred regimens of amoxicillin-clavulanate (amox/clav) or doxycycline for a 5-day duration are not being utilized. An electronic health record (EHR) best practice alert (BPA) aims to enhance prescribing efficiency and adherence to optimal practices. This study evaluates the effectiveness of an EHR BPA in improving outpatient sinusitis prescribing patterns.

**Figure:**
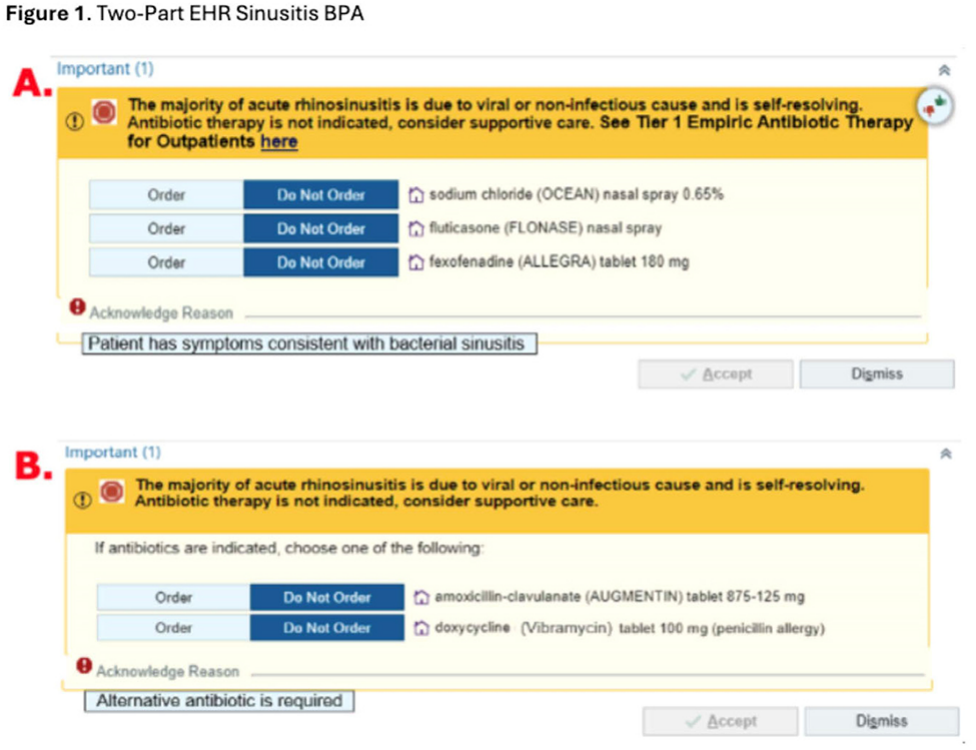
A. Part A of the EHR sinusitis BPA provides supportive care options to choose from with the option to proceed with no antibiotic prescription or to acknowledge that the “Patient has symptoms consistent with bacterial sinusitis” leading to part B. B. Part B displays the preferred or optimal bacterial sinusitis antibiotic prescriptions with a 5-day treatment duration pre-populated. The prescriber can also not order these preferred regimens and select that an “Alternative antibiotic is required”.

**Figure:**
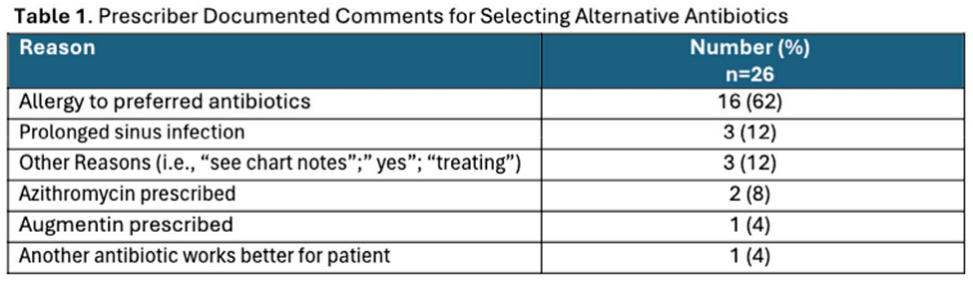

**Methods:**

IRB-approved, cross-sectional study examined adult outpatient encounters with an acute sinusitis diagnosis from 3/1/25-4/29/25 at outpatient clinics in southeast Michigan. The sinusitis BPA was integrated into the EHR on 3/1/25 [Figure 1]. An antibiotic prescription was defined as optimal if an antibiotic was prescribed using the BPA that defaults to amox/clav or doxycycline for a duration of 5-days [Figure 1b]. The primary outcome was the proportion of optimal antibiotics prescribed for sinusitis with an accepted BPA recommendation. Secondary outcomes were median time taken by the prescriber to acknowledge the BPA, which antibiotics were prescribed, and the proportion of prescribers that documented a comment if an alternative antibiotic was required.

**Figure:**
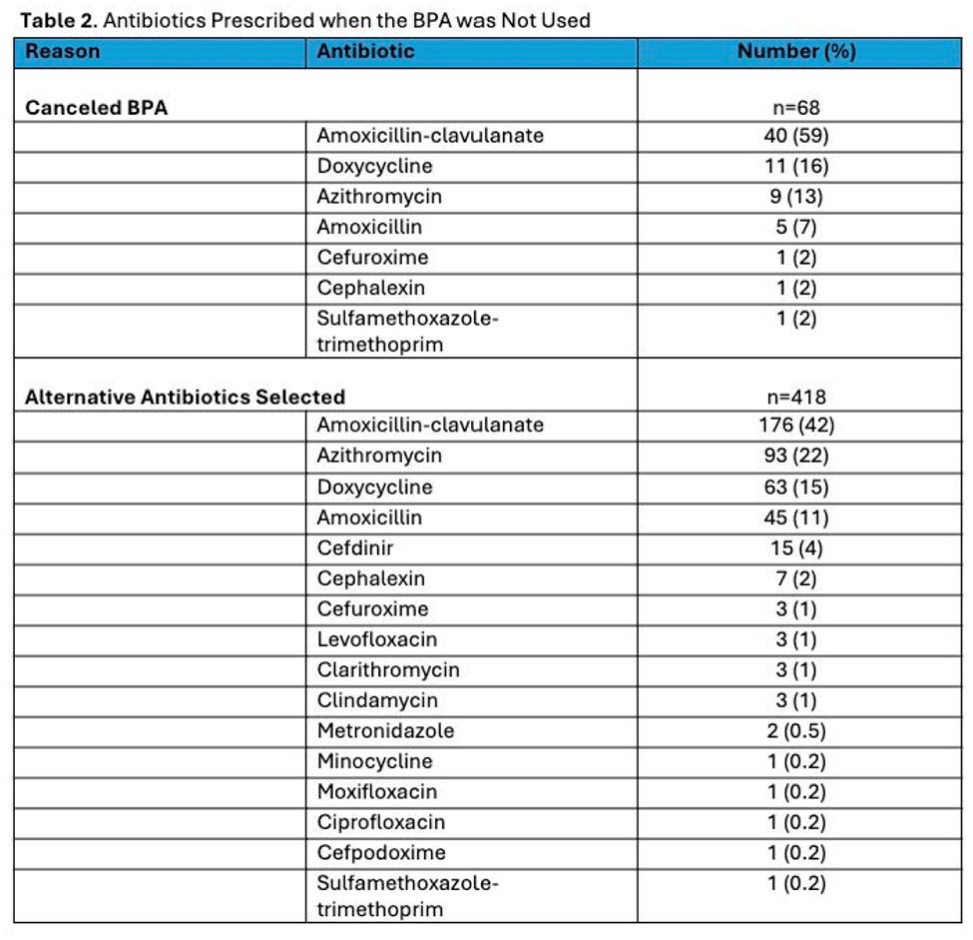

**Results:**

The EHR BPA triggered 905 times. When the BPA was accepted, there were 190 (21%) optimal prescriptions (174 amox/clav and 16 doxycycline). The median (IQR) time to acknowledge the BPA was 11 (6-17) seconds. In cases of non-BPA acceptance, alternative antibiotics were required in 418 encounters (46%), with reasons documented in comments by 26 prescribers (6%) [Table 1] and 68 prescribers (8%) canceled the BPA advisory. Antibiotics prescribed when the BPA was not accepted are listed in Table 2. Additionally, 229 encounters had no documentation of why the BPA was not accepted where amox/clav (109, 48%) and doxy (26, 11%) were prescribed most often with durations of 7-14 days.

**Conclusion:**

Implementing a tailored prescription strategy into the EHR led to 21% of outpatient sinusitis encounters receiving optimal antibiotic treatment. Enhancing EHR utilization and prescriber familiarity with EHR optimization are crucial for improving sinusitis care.

**Disclosures:**

John R. Craig, MD, Aerin Medical, Inc: Advisor/Consultant

